# A Study on the Quality of Patient-Centred Endometriosis Mobile Applications: Analysis and Future Prospects

**DOI:** 10.1155/ogi/5582127

**Published:** 2025-04-15

**Authors:** Kevin Dominique Tjandraprawira, Edwin Armawan, Muhammad Alamsyah Aziz, Tono Djuwantono

**Affiliations:** ^1^Department of Obstetrics and Gynecology, Faculty of Medicine, Dr. Hasan Sadikin General Hospital, Universitas Padjadjaran, Bandung, Indonesia; ^2^Bandung Fertility Center, Limijati Women and Children Hospital, Bandung, Indonesia

**Keywords:** applications, endometriosis, patient-centred apps

## Abstract

**Introduction:** Female technology or ‘femtech' is the availability of mobile applications (apps) to monitor specific aspects of women's health. It touts the benefits of technology to empower women with regards to their health, while also allowing close collaboration between patients and physicians. Endometriosis-specific apps is a channel by which female patients discover their endometriosis diagnosis. However, there is currently a paucity of high-quality and evidence-based endometriosis apps. This study assessed the current state and overall quality of such apps.

**Materials and Methods:** This retrospective study assessed patient-centred endometriosis apps on the Apple iTunes Store, in January 2023, excluding certain categories such as conference guidance, gaming, private healthcare, and clinical trial apps. The key term “endometriosis” was used. All patient-centred apps were included. The apps were assessed using the APPLICATIONS scoring system, considering objective factors like pricing, subscriptions, literature references, in-app purchases, connectivity, advertisements, search fields, interoperability, and subjective elements such as navigation ease and presentation. The maximum score is 16 and all applications were assessed in English.

**Results:** Out of the initial 22 apps, 12 (54.5%) were excluded, leaving 10 (45.5%) for analysis. Most apps lacked comprehensiveness, but two apps (LUNA-endometriosis and Frendo) stood out with high scores. These apps incorporated certified scoring systems, provided recommendations for tests and follow-up visits, and offered evidence-based literature on endometriosis. The remaining apps scored poorly, focusing on alternative treatments, healthy diets, or functioning solely as symptom diaries, with limited information on diagnosis and management. Only a few apps allowed booking follow-up visits, and most lacked the capability to export patient-specific data. Few apps measured the likelihood of endometriosis whilst others relied on previous diagnoses. The majority of apps were free, whilst in-app purchases centred around alternative treatments. Overall, the apps were user-friendly, featuring vibrant colours and intuitive interfaces.

**Conclusions:** Only two patient-centred endometriosis apps scored highly in the APPLICATIONS scoring system as they were high-quality, evidence-based and incorporated valid medical recommendations. Other applications scored low as their recommendations lacked quality scientific evidence.

## 1. Introductions

Health-specific applications are increasing both in number and popularity in the 21^st^ century [1]. The apps cover almost all aspects of life, from monitoring food consumptions, calorie intakes, predicting menstrual cycles and even act as a safeguard against child abuse [2–4]. Specifically, there is a gain in popularity of using health-specific applications (apps) in women's health [5]. This coincides with the ubiquitous adoption of smartphones. The rise of female technology or ‘Femtech' is the availability of mobile apps to monitor various aspects of women's health and it is increasing in popularity [6, 7]. Proponents of Femtech argue that such apps empower women to retain greater control of their health [8]. Others argue that apps are useful in allowing collaboration between patients and physicians [5].

Femtech provides ample opportunity to develop female disease-specific apps, including endometriosis [7]. Endometriosis affects 10%–15% of reproductive age women and up to 70% of those with chronic pelvic pain [9]. Endometriosis-specific applications may serve as their first experience of discovering endometriosis, learning its signs and symptoms and trialling conservative and/or alternative treatments. The apps may have a plethora of capabilities, ranging from simple questionnaire-based apps that attempt to self-diagnose endometriosis to complex apps that provide evidence-based recommendations that are useful for patients. However, there is no standardisation and rigorous quality control on the development of endometriosis-specific apps.

Currently, there are only a small number of endometriosis apps accessible to the public. Whilst developers vary in their objectives, the apps are either patient-centred or healthcare provider-centred. A previous study by Shaia et al. only identified 7 apps useful for reproductive endocrinology and infertility specialists [5]. A more recent study by Moumane and Idri has identified more apps regarding endometriosis [10]. However, the quality of the apps was noted to be quite low, especially the Android apps [10]. They found very few applications that would cover all aspects of endometriosis, from diagnosis to complete management [10, 11]. They also pointed out the need for such apps to treat patient data as classified medical records with adopting advanced encryption strategies and storing such data securely in dedicated servers [11].

There are no studies yet on patient-centred endometriosis apps, both regarding their quantity and quality. This is a gap that we aim to bridge by evaluating the available apps and their utility in allowing collaboration between patients and clinicians.

## 2. Materials and Methods

This is a retrospective study on all endometriosis-specific apps available on the Apple iTunes store. A search of the online store was done in 20–23 January 2023 and a total of 22 apps were identified. The key term “endometriosis” was used to identify apps. The search was done on the Indonesian iTunes store as this is where the authors are based. Patient-centred apps were those apps using patient-centred language and few nonmedical jargons and for this study, all patient-centred apps were included. The exclusion criteria were the following:

1. Electronic versions of textbooks on endometriosis
2. Guidance apps for specific conference on endometriosis
3. Gaming apps
4. Private office or hospital-acquainted apps
5. Professional association apps
6. Clinical trial apps
7. Medical device accompanying apps

The apps were ranked according to the APPLICATIONS scoring system as described by Shaia et al. [5, 12] This scoring system was devised for assessing mobile applications and the original authors utilised the scoring system for their study on assessing the quality of applications in Obstetrics and Gynecology [12]. Tables 1 and 2 describe the scoring system. The scores were tabulated, and the apps were ranked. The apps comprehensiveness score was deduced from the app's capability to participate in patient clinical decision-making. Ease of navigation and subjective presentation were evaluated on a Likert scale with scores between 1 and 5 (1 being poor, 2 below average, 3 average, 4 more than average and 5 excellent). The authors KDT and TD independently reviewed each app and allocated scores for the apps. The maximum score according to the APPLICATIONS scoring system is 16. All applications were assessed in English.

This study is exempt from the Institutional Review Board and did not require any ethical approvals as this study does not involve any risk to human subjects.

## 3. Results

The initial search on the Apple iTunes Store identified 22 apps. 12 apps were excluded based on the exclusion criteria above. 10 apps were included in this final analysis.  Table 3 lists the scores of each app. Apps' features are graded according to the APPLICATIONS scoring system. An integral feature is the app's comprehensiveness for clinical decision making. The two highest-scoring are LUNA-endometriosis and Frendo with 12 points each from a possible maximum score of 16. LUNA-endometriosis has the CE (Conformité Européenne)-certified ENDOscore questionnaire that calculates the likelihood of one's risk of having endometriosis. This is a validated questionnaire and capable of recommending follow-up tests to diagnose endometriosis. The other app is Frendo which has a similar survey. It has a wide collection of evidence-based articles and information regarding endometriosis sourced from Endometriosis Australia organization. In addition, from a qualitative perspective, the abundance of images and videos in both apps also improved the user experience.

The other apps scored low in the APPLICATIONS scoring system due to their lacking in various aspects. First, only a few provide functions that measures/calculates someone's risk of suffering from endometriosis. Whilst the app LUNA-endometriosis has a CE-certified questionnaire, many others employ surveys relying on a previous endometriosis diagnosis. Thus, the utility of the latter group in establishing a likely diagnosis of endometriosis or recommending a follow-up medical consultation was little. Another app is apparently an extension to a private clinic. Nezhat Endometriosis is an app that gives a survey on the likelihood of one suffering from endometriosis. Upon filling the survey, users are given little information about their disease and immediately redirected to the booking page of a private fertility clinic and the app serves as a screening tool for the surgery.

Second, very few apps based their contents on evidence-based literature. On one hand, a number of apps chose to emphasise on recommending complementary and alternative treatments, including hypnosis, yoga, relaxation techniques and breathing works, such as Endometrix and Relieved. Users are expected to list their preferences and traits, subsequently used to offer in-app purchases offering tailored treatment programs. On the other hand, some apps focus solely on diets. Apps such as Endometriosis+ and Living with Endometriosis focus on providing recommendations for healthy diets and personal recipes. The apps list the ingredients claimed to provide benefits against endometriosis. Unfortunately, the recommendations and recipes are not evidence-based and do not include references to a specific literature.

Third, other apps only function to record symptoms of endometriosis without offering further information on timely diagnosis and management. Apps such as ENdi, Endometriosis Diary and myFLO focus on providing a diary of endometriosis symptoms. The diary function is diverse with some apps preferring a list-based approach where users can tick the symptoms that match their experiences. The list can get very detailed with meticulous descriptions of each symptom. Other apps adopt a different approach, preferring a free-text approach to their diaries, allowing patients to use their own words to describe their symptoms.

Fourth, only 2 apps (LUNA and Frendo) explicitly cite their resources. Apps that offer alternative forms of treatment to endometriosis offer very little to no references/literature regarding the efficacy of their recommendations.

Fortunately, most of the apps are free and tend not to require any paid subscriptions nor in-app purchases. In fact, the two highest scoring apps are free with no subscriptions and no in-app purchases. However, the apps that require subscriptions and in-app purchases operate on the basis that users can unlock specific programs and/or treatment plans tailored to one's preferences. Most often, they are apps offering alternative forms of treatments for endometriosis.

The apps in general are well-designed. Most of the apps are easy to use and their presentations combined vibrant colours, intuitive user interface coupled with attractive designs for users. However, some apps do require updates as some apps have compatibility issues with the latest Apple's operating system, disallowing some features to appear whilst also causing the apps to crash when certain functions are pressed. Furthermore, some apps have not been updated for some time and despite the developers' promise on the app's about page, their promises are still to be fulfilled.

## 4. Discussion

This study was done to gain an overview of the applications that are available for endometriosis sufferers and to evaluate if the apps offer evidence-based and accurate literature for those suffering from endometriosis.

Endometriosis is known to affect about 10% of females worldwide with a higher prevalence among women suffering from chronic pelvic pain and/or infertility [13]. However, a search of the Apple App Store has revealed a very limited number of patient-centred apps for endometriosis. A search in January 2023 discovered only 10 apps that can be classified as trying to provide patient support for endometriosis. Contrary to some previous studies that investigate apps that are health provider-centred, this study looks at patient-centred apps [5].

It is alarming that out of the 10 apps that we could find on the App store, only two provided are evidence-based regarding diagnostics and recommended treatments. Whilst the two apps provided articles and information referencing medical literature, the other apps provided little or no literature. This is especially true for the apps offering alternative forms of treatment for endometriosis. Furthermore, our assessment on the companies behind the apps provided a less-than-ideal finding as all apps did not involve reproductive endocrinologists or endometriosis physicians in their team of developers. The recurring story has been fellow sufferers of endometriosis concerned by the lack of available apps that decided to create apps of their own from their personal medical journey. Unfortunately, this is not unique to the field of obstetrics and gynaecology as apps that aim to help patients manage mental-health issues also suffer from the same problem. Research by Marshall et al. described that only < 4% of mental health apps investigated had research to justify their claims on efficacy and effectivity [14]. The same finding has also been repeated in the field of dermatology, as few apps had medical expert involvement in their developments [15]. Even fertility-tracking apps are mostly unreliable as they rely on inaccurate algorithms [4]. This is worrying as the lack of evidence behind these apps disallows them from being an effective tool for patient-physician partnership and in the case of fertility-tracking apps, barring them from being an effective mode of contraception [4, 16].

Some apps are focused on complementary and alternative medications (CAM) for endometriosis. There are various forms of CAM offered, such as yoga, meditation, hypnosis and breathing works. However, focusing on CAM for endometriosis whilst ignoring the medical and surgical therapies for endometriosis is inappropriate. Mira et al. reported in their meta-analysis that only acupuncture is shown to provide a significant benefit in pain reduction amongst sufferers of endometriosis, whilst other forms of CAM, including yoga have not shown to produce significant benefits [17]. Their status as CAM often causes them to be regarded as adjuncts to definitive medical therapy and they are not known to cure endometriosis [17]. If the apps focus on promoting only certain forms of CAM whilst discounting the definitive medical therapies, they may preach the wrong message to the general public about endometriosis and its treatment.

Dietary modification and endometriosis are a rapidly developing topic. Studies recommending the adoption of Mediterranean and/or gluten-free diets have been published [18, 19]. The popularity of apps recommending tailored diets and recipes for endometriosis patients benefits the patients. However, the apps should be clear that dietary modifications should not form the main pillar of endometriosis treatment and should only serve as an adjunct to definitive medical therapies [18]. Furthermore, it would be better if the apps cited the literature/references backing their claims on the benefits of their tailored recipes/dietary recommendations.

The embedded diary function is pivotal for these apps, as users can log their symptoms daily. The free-text approach in some apps allows the users to be more emotive in their accounts but lacks the ability to be systematically assessed. The questionnaire-like interface in others allows more opportunity for comprehensive study but some users may feel constrained whilst reporting their symptoms if the lists do not match the users' experiences.

There are contrasting views on the influence of patient-generated health data (PGHD) towards patient care. A popular view, advocated by apps developers, is that PGHD allows greater patient autonomy and better collaboration between patient and clinicians as it allows patients to present real-time data to be reviewed by clinicians during their consultations [20]. They also argue that symptom logs allow clinicians to review additional symptoms and patient data that may not appear during patient consultation [20]. However, real-life experiences have presented issues with this claim. Whilst clinicians may argue that the apps promote self-awareness that enhance patient self-care, the lack of uniformity in symptoms and data reporting has led to technical barriers and reluctance among clinicians to adopt this approach [20, 21]. The lack of ISO certification and common approach in app development also contribute to the wide gap in app usability [10, 11]. The older generation physicians may not be as technologically-savvy as the younger generations and reviewing PGHD may lead to information overload that adds further burden to the tight clinic appointment time constraints. Other clinicians have noted that reviewing PGHD is not like receiving a patient's account of their symptoms in person [20]. The emotive nature of a patient's symptoms may be lost if an emphasis is made on primarily reviewing PGHD. Furthermore, apps focusing on complementary and alternative treatments are not designed for collaboration with clinicians.

An important aspect to consider for future mobile apps development is the safety of patient health data. A feature of the iOS apps is the integration of authentication to use the app's functionalities. Most iOS apps have taken advantage of the in-built user identity authentication function in the mobile device to protect the access and guarantee the safety of patient health data [10]. As pointed out by Moumane and Idri, the international standard IS13485 regarding quality management system (QMS) is the benchmark to which all apps should adhere to, with regards to the design of medical devices domain [10, 11, 22]. A secure and encrypted health data storage (HDS) system is also a prerequisite for quality medical applications, including for mobile endometriosis applications [10, 11, 22]. From our findings, the app developers did not specifically mention the more detailed safety approaches to be undertaken with regards to the storing and processing of confidential patient data. Such statements should be more standardised and should be included in future application developments to ensure that patient data are stored safely and in line with the latest data protection laws, such as the European Global Data Protection Regulation (GDPR).

The above findings clearly demonstrate is that there is abundant opportunity for app developers to develop high-quality patient-centred evidence-based endometriosis apps. Two apps scored highly in our assessment, LUNA Endometriosis and Frendo and other developers should aim to improve on this. We suggest that an optimum patient-centred endometriosis app should include a toolkit that enables a common layperson user with chronic worsening dysmenorrhea to receive a differential diagnosis of endometriosis, receive suggestions of subsequent diagnostic studies and consultations with a consultant. The apps should also be available at an affordable price with interplatform compatibility (iOS and Android). They should offer various multimedia contents (photos, videos) and provide evidence-based content with appropriate citation/references. The app developers should also include endometriosis physicians in their app development to better understand the requirements of both patients and physicians. The apps should also be intuitively easy to navigate with vibrant, attractive designs. Furthermore, the apps should undergo certification after assessing mobile usability models, like ISO 9126, that provide structured evaluation metrics [11]. As described at length by Moumane et al. (outside the scope of this article), a standardised certification of mobile applications, including endometriosis-related applications would improve user experience across all platforms [11]. LUNA Endometriosis and Frendo have accomplished many aspects well but with room for improvement.

A strength of this study is its simplicity. A literature search has revealed that no other study has investigated the utility of patient-centred endometriosis apps in supporting care and treatment of endometriosis alongside healthcare professionals.

This study has several limitations. First, the applications list is continuously updated with new applications coming and older ones going phased out from the online app store. Secondly, the applications themselves may be updated after the publication of this research. Thus, the findings of this study in January 2023 may no longer be wholly accurate by the time of publication.

The authors had performed a similar search on the Google Play Store for Android smartphones. However, the search identified similar applications with the Apple store and there were some applications on Apple store that were not available on Google Play Store. Thus, the authors decided to focus the search on Apple App Store. This is a similar finding reported by Moumane and Idri, with their research highlighting the inferior quality of the Android apps as opposed to iOS apps [10].

## 5. Conclusion

Only two endometriosis apps present high-quality evidence-based information to be used in conjunction of medical care of endometriosis. Other applications scored low as their recommendations lacked quality scientific evidence. Applications should enable the users to assess if they were at risk of having endometriosis and/or needed referrals and should provide users more evidence-based recommendations and contents. Future developments should involve physicians more in the app design and that certification should be pursued to elevate the user experience and reduce gaps in mobile application usability.

## Figures and Tables

**Table 1:** Applications scoring system and weights, reproduced from Shaia et al. [5].

Component	Score	Description
App comprehensiveness	3	See Table 2
Price	1	0 = priced, 1 = free
Paid subscription	1	0 = required, 1 = not required
Literature used	1	0 = No references, 1 = references used
In-app purchase	1	0 = present, 1 = absent
Connectivity	1	0 = internet required; 1 = internet not required
Advertisements	1	0 = present, 1 = absent
Text search field	1	0 = no search field, 1 = search field present
Interplatform compatibility	1	0 = iOS device or android tablet; 1 = iOS device and android tablet

*Other components*		
Images/figures	1	0 = present, 1 = absent
Videos	1	0 = present, 1 = absent
Special features	1	0 = present, 1 = absent
Navigation ease	1	0 = ease of navigation score < 3, 1 = ease of naviagation score ≥ 3
Subjective presentation	1	0 = subjective presentation score < 3, 1 = subjective presentation score ≥ 3
Total	16	

*Note:* App, application.

**Table 2:** Apps comprehensiveness scoring criteria, reproduced from Shaia et al. [5].

Clinical decision-making criteria	Description
Clinical decision support systems	Active knowledge system that uses two or more items of patient data to generate case-specific advice
Clinical treatment guideline	Recomends how to treat a specific disease process
Disease diagnosid aids	Constellation of symptoms focused to one diagnosis
Differential diagnosis aids	Constellation of symptoms proviidng you with a range of diagnoses
Medical calculators	Provides a numerical output with one or more inputs
Laboratory test ordering	Recommends laboratory tests for initial workup of a symptom(s)
Laboratory test interpretation	Clarifies laboratory test result
Medical examinations	Physical examination aid

*Note:* A score of 0 was assigned if the app had no clinical decision-making capacity. A score of 1 was assigned if the app included 1–2 criteria. A score of 2 was assigned if the app included 3–5 criteria. A score of 3 was assigned if the app included 6–8 criteria.

**Table 3:** Applications scoring system.

Criteria	App comprehensiveness	Price	Paid subscription	Literature used	In-app purchase	Connectivity	Advertisements	Text search field	Interplatform compatibility	Other components	Navigation ease	Subjective presentation	Total
Endometriosis +	1	0	0	0	0	0	1	0	1	0	0	0	3
MyFLO period tracker	0	0	1	0	1	0	1	1	0	1	1	1	7
Nezhat endometriosis advisor	1	1	0	1	0	0	1	0	1	0	1	1	7
Living with endometriosis	0	1	1	0	1	0	0	1	0	1	1	1	7
Endometriosis diary	0	1	0	0	1	1	1	1	1	1	1	0	8
LUNA - endometriosis	2	1	1	0	1	0	1	1	1	2	1	1	12
ENdi: track your endometriosis	0	1	0	0	0	0	1	1	0	0	1	0	4
Relieved: period pain hypnosis	0	1	0	0	0	0	0	0	0	1	1	1	4
Endometrix	0	1	0	0	0	0	1	0	1	1	1	1	6
Frendo	1	1	1	1	1	1	1	0	1	2	1	1	12

## Data Availability

Research data are available to the authors upon reasonable written request.
